# Silver Nano/Microparticles: Modification and Applications 2.0

**DOI:** 10.3390/ijms21124395

**Published:** 2020-06-20

**Authors:** Xuan-Hung Pham, Jaehi Kim, Bong-Hyun Jun

**Affiliations:** Department of Bioscience and Biotechnology, Konkuk University, Seoul 143-701, Korea; phamricky@gmail.com (X.-H.P.); susia45@gmail.com (J.K.)

Currently, nano/microparticles are widely used in various fields [1,2,3]. Silver particles are one of the most vital materials among the various particles, due to their unique optical-physical-chemical properties. The materials have been proposed for various fields, such as bio-sensor, diagnostics, imaging, catalyst, solar cell, and antibacterial [4,5,6,7,8,9,10,11,12,13,14]. In particular, size-dependent unique plasmonic properties make the particles superior in biomedical applications [15,16,17,18,19,20]. 

Due to this importance of silver materials, the first version of “silver nano/microparticles: modification and applications” was successfully published last year with 10 outstanding papers [21,22,23,24,25,26,27,28,29,30]. This version 2.0 of the Special Issue also provides original contributions detailing the synthesis, modification, and applications of silver materials. Eleven outstanding papers which describe examples of the most recent advances in silver nano/microparticles are included.

The plasmonic properties of silver nanoparticles have been applied to the detection of harmful substances based on surface-enhanced Raman scattering (SERS), due to its non-destructive, rapid, molecular fingerprinting and ultrasensitive and photostable properties [31]. Because histamine intoxication associated with seafood consumption can cause illnesses, Kim-Hung et al. reported facile histamine detection by SERS using a plasmonic silver-gold nanostructure [32]. They successfully detected histamine with SERS using the nanostructure (3.698 ppm LOD). Pham et al. reported the sensitive and quantitative detection of pesticides based on SERS by using an internal standard containing nanostructures [33]. For the study, 4-mercaptobenzoic acid labeled silver-gold nanoparticles were used for a sensitive and quantitative thiram detection, and a range of 240 to 2400 ppb with a detection limit of 72 ppb of thiram was detected.

Silver nanoparticles have great potential as an antibacterial agent. Nakamura et al. reviewed the synthesis and application of silver nanoparticles for the prevention of infection [34]. In particular, they focused on environment friendly synthesis and the suppression of infections in healthcare workers. Nakamura et al. reported that ultraviolet irradiation enhances the microbicidal activity of silver nanoparticles via hydroxyl radicals [35]. They showed that UV irradiation to silver nanoparticles is effective for enhancing their microbicidal activity, due to the antimicrobial activity of reactive hydroxyl radicals which were generated from silver nanoparticles by UV irradiation. The UV irradiation-mediated enhanced production of reactive hydroxyl radicals is generated rapidly from silver nanoparticles. Silver nanowires, which exhibit excellent conductive properties, have been intensively studied for thermal and electronic applications. Mori et al. evaluated the antibacterial and cytotoxicity properties of silver nanowires and their composites with carbon nanotubes for biomedical applications [36]. Li et al. reported a simple, sustainable, and environmentally friendly method for the in situ fabrication of silver nanoparticles in mesoporous TiO_2_ films decorated on bamboo via self-sacrificing reduction to synthesize nanocomposites with an efficient antifungal activity [37]. The composite films-endowed bamboo exhibited an excellent antifungal activity to T. viride and P. citrinum. Because of the high biocompatibility, low cost, and ease of manufacture of the poly(methylmethacrylate) (PMMA) resin, it is widely used in medical and dental fields. Matteis et al. reported that silver nanoparticles added a poly(methyl methacrylate) dental matrix for topographic and antimycotic studies [38]. 

Since silver nanoparticles are attractive alternatives to plasmonic gold nanoparticles, the controlled synthesis of metal nanoparticles with a defined morphology can be important for such fields as biochemistry, catalysis, biosensors, and microelectronics. Cyclophanes, which have a variety of cyclophane 3D structures and unique redox abilities, can create metal nanoparticles. Padnya et al. summarized the recent advances in the synthesis and stabilization of Ag (0) nanoparticles based on self-assembly of associates with Ag (I) ions with the participation of cyclophanes [39].

Biological molecules have potential for the synthesis of metallic nanoparticles as green and economic methods. Tanaka et al. reported the green synthesis of silver nanoparticles by using peptides [40]. They used array-based screening to identify a list of mineralization peptides with various physicochemical properties. They evaluated the silver nanoparticle mineralization activity of the top 200 gold nanoparticle-binding peptides, and the highest silver nanoparticles synthesis activity was shown in the presence of EE and EXE peptides (E: glutamic acid, and X: any amino acid).

Silver islands films (SIF) can play an important role among plasmonically active platforms. Szalkowski et al. reported silver islands substrates which prepared on demand based on the laser-induced photochemical reduction of silver compounds on a glass substrate [41]. The prepared SIF showed a strong plasmonic activity. 

Hybrid systems of photosynthetic pigment–protein complexes with plasmonically active metallic nanostructures can be a useful design for future biomimetic solar cells. Kowalska et al. reviewed SIF for enhancing light harvesting in natural photosynthetic proteins [42]. They presented the results of a variety of photosynthetic complexes upon coupling with SIF structures.
